# The efficiency of electronic list-based multidisciplinary team meetings in management of gastrointestinal malignancy: a single-center experience in Southern China

**DOI:** 10.1186/s12957-018-1443-1

**Published:** 2018-07-19

**Authors:** Yujie Yuan, Jinning Ye, Yufeng Ren, Weigang Dai, Jianjun Peng, Shirong Cai, Chuangqi Chen, Min Tan, Wu Song, Yulong He

**Affiliations:** 1 Center of Gastrointestinal Surgery, The First Affiliated Hospital, Sun Yat-Sen University, 58, 2nd Zhongshan Road, Guangzhou, 510080 Guangdong Province People’s Republic of China; 2 Department of Radiation Oncology, The First Affiliated Hospital, Sun Yat-Sen University, Guangzhou, People’s Republic of China; 3 Center of Gastric Cancer, Sun Yat-Sen University, 58, 2nd Zhongshan Road, Guangzhou, 510080 Guangdong Province People’s Republic of China

**Keywords:** Gastrointestinal malignancy, Multidisciplinary, Electronic checklist, Efficiency

## Abstract

**Background:**

The multidisciplinary team (MDT) discussion has earned increasing popularity for the delivery of cancer care. However, MDT meeting (MDTM) is time and resource intensive, and some efforts to optimize discussion processes are required. This study aims to investigate the efficiency of electronic list-based MDTM in treatment of gastrointestinal (GI) malignancy.

**Methods:**

Between January 2015 and December 2016, patients with GI cancers were retrospectively reviewed. Patients permitting an MDTM with our novel technique (eMDT group) were compared with those undergoing a traditional discussion (cMDT group). The efficiency of MDT working, including time cost per meeting or case and overall number of reviewed cases, was checked, with accuracy of clinical staging and other outcomes explored meanwhile.

**Results:**

Three thousand six hundred seventy-four patients were included, with 2156 (58.7%) and 1518 (41.3%) cases for eMDT and cMDT groups, respectively. Comparisons in age (*P =* 0.529), gender (*P =* 0.844), cancer type (*P =* 0.218), treatment plan (*P =* 0.737), and pathological stage (*P =* 0.098) were not significant between groups. However, the average time cost in both each meeting (149.4 vs. 205.1 min; *P <* 0.001) and each case (3.1 vs. 6.2 min; *P <* 0.001) was markedly reduced. Besides, this novel technique was associated with improved accuracy of clinical staging (*P =* 0.070) and reduced hospital stay (*P <* 0.001) compared with the traditional approach, with similar incidence of complications observed (*P =* 0.243).

**Conclusions:**

The MDT working based on an intelligent checklist could save considerable time while not affecting treatment of GI malignancies. The improved efficiency also earns an increased capacity of hospital admission and in-patient care.

## Background

Gastrointestinal (GI) malignancies, such as esophago-gastric cancer, colorectal cancer, and gastrointestinal stromal tumor (GIST), remain a great health burden in China [1]. To ensure the best care for oncologic disease, a multidisciplinary approach has been established since 1975 [2]. However, multidisciplinary team (MDT) working has only been effective in clinical practice since the late 1990s [3]. Meanwhile, its efficiency and efficacy in management of various cancers have been questioned due to intensive time and resources spent on this process [4, 5].

In general, an MDT should consist of several specialized experts on oncologic fields working together for specific cancer [6–8]. This team often meets at a fixed time to discuss the diagnosis and treatment of newly referred or admitted patients with complex diseases, and commonly summarize a universal plan based on current guidelines and individual experience. Numerous evidence has confirmed that MDTs achieve better adherence to guidelines, better diagnostics, and better adherence to formulated treatment plans [9–13].

Over the last two decades, the multidisciplinary approach, commonly implemented as an MDT meeting (MDTM), has become a routine procedure for managing most surgical patients in our center. To our knowledge, the process of decision making during the MDTM is time-consuming, which is mainly dependent on the quality of preoperative materials for discussion and complexity of cancer disease. To improve the efficiency of MDT working, an electronic checklist designed for weekly MDTM was established recently in our center. It was applied to the discussion process to replace the conventional case report. This electronic technique could visualize clinical data and optimize waiting time during the MDTM. This study aimed to investigate the efficiency of this novel method in management of GI malignancies.

## Methods

### Patient population

The current study was a single-center retrospective analysis at a tertiary teaching hospital in Southern China. All data were prospectively collected from our designed database for patients with gastrointestinal cancer. The study protocol was approved by the Institutional Review Board of our hospital. Written informed contents were obtained from all subjects and their relatives before any treatment.

**Fig. 1:**
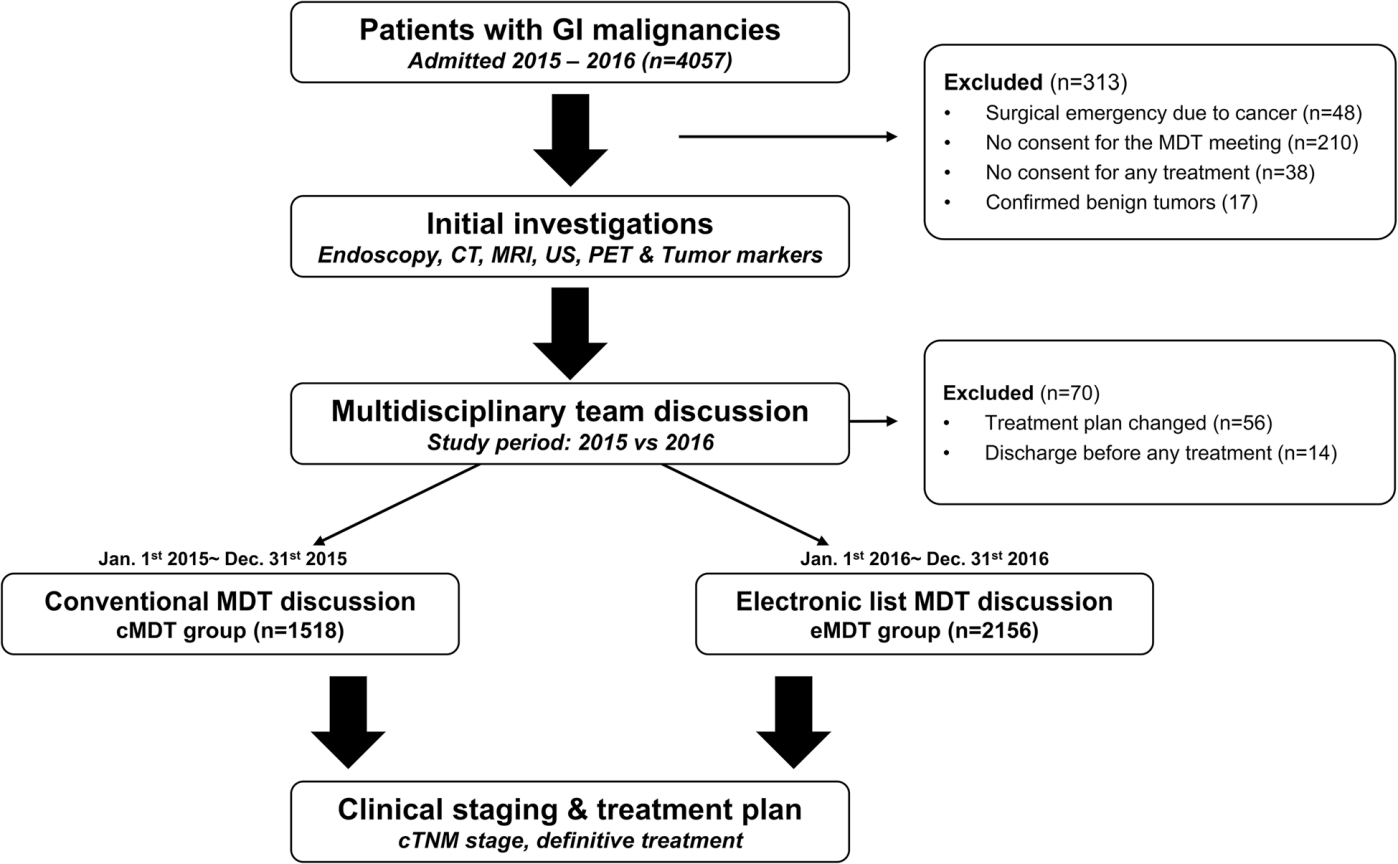
Flow chart of this study. In the study period of 2015, patients received a traditional MDT meeting for clinical assessment and treatment; while in 2016, patients received our novel electronic checklist during the MDTM for clinical management

From January 2015 to December 2016, patients referred or admitted to our unit for cancer treatment were first enrolled from our database. Those who did not join the MDT discussion were excluded from the final analysis. In details, patients with histologically confirmed carcinoma of GI tract were eligible for the MDTM and were divided into two groups according to time intervals when the meetings hold. Briefly, patients who attended an MDTM within the first year of study period (from 1 January 2015 to 31 December 2015) were assigned to the conventional MDT (cMDT) group, since they were discussed with traditional procedures [14]. Those who had a checklist-based MDTM in the second year of study period were assigned to the electronic MDT (eMDT) group. Of note, initial staging investigations, which included computed tomography (CT) scans, endoscopic examination, biopsy pathology, and additional magnetic resonance imaging (MRI) or endoscopic ultrasonography (EUS) if required, were routinely performed for all patients before the MDTM. A flowchart of the study design is presented in Fig. 1.

### Electronic list-based MDT discussion

Before 2016, included patients were discussed with a conventional method, which consisted of case report by resident, case summary and disease assessment by attending surgeon, review of radiological and pathological results by specific experts, and treatment suggestions by all members joining the meeting. In practice, all processes were completed in sequence during the meeting, with none of them skipped before any treatment.

**Fig. 2:**
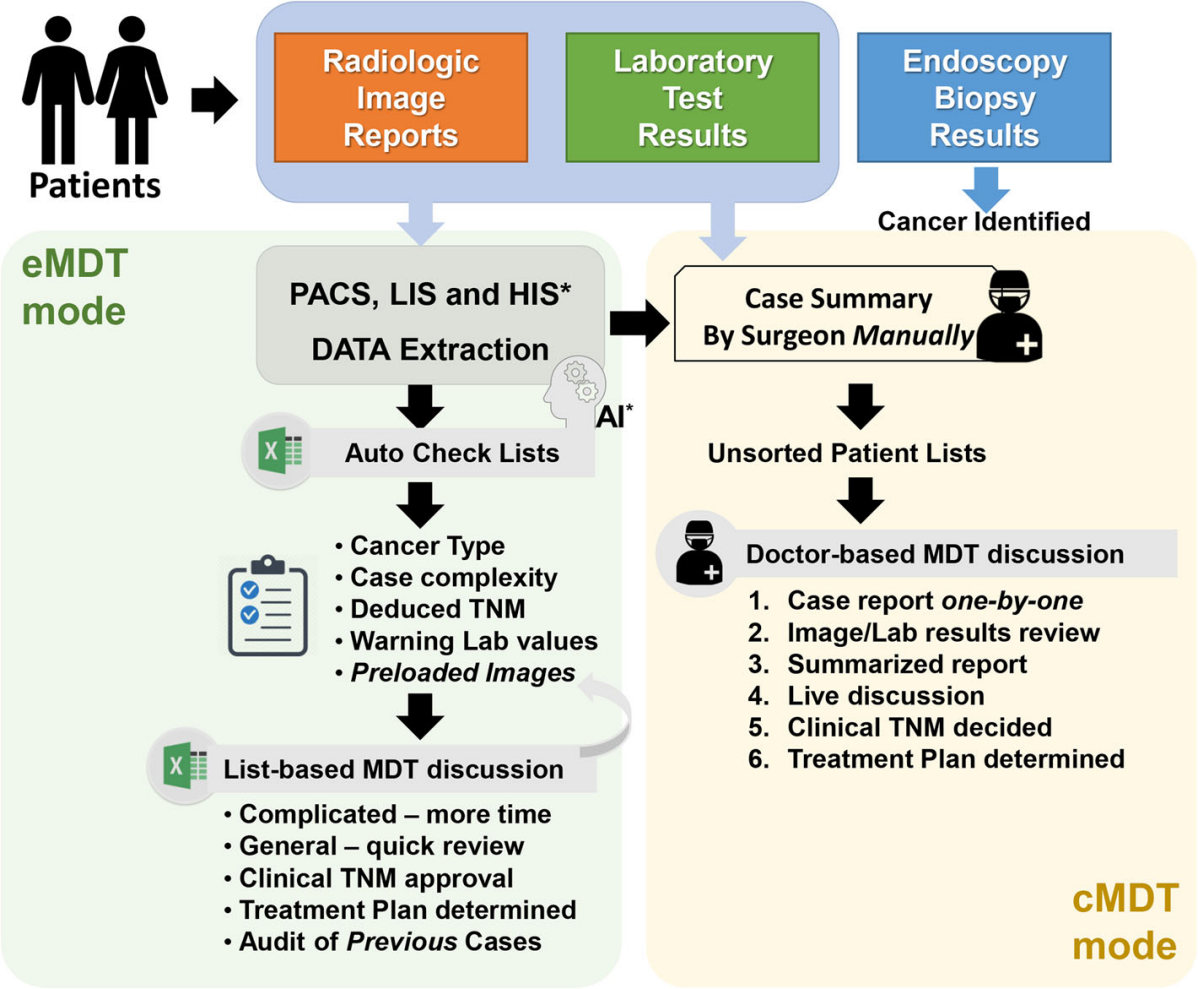
Comparisons of procedures between eMDT and cMDT meetings in management of gastrointestinal malignancy. A spreadsheet produced by EXCEL was employed for quick case review and MDT discussion in the eMDT group. PACS, picture archiving and communication systems; LIS, laboratory information system; HIS, hospital information system; AI, artificial intelligence (computing based on pre-defined program/rule)

To improve the efficiency of discussion and decision-making processes, an intelligent spreadsheet, which was designed by using Microsoft© EXCEL software (version 2016, Microsoft Corporation, Redmond, WA, USA), was utilized to collect clinical data from our electronic medical records (Fig. 2). In brief, valuable data including positive laboratory results, important radiologic evaluation for regional and distant metastatic disease, and basic comorbidities were extracted automatedly from our digital systems by prospectively defined rules. After that, those data were employed to deduce an objective description of oncologic status, especially for tumor staging, with a form of sortable case list in the EXCEL. Finally, all produced case lists formed an electronic checklist for a weekly MDTM.

Patients with a formulated summary of current disease would be listed together by sorts of age, cancer type and complexity. Of note, a predicted clinical stage for each patient, which was approved by attending surgeons before the meeting, would be attached to the checklist. Importantly, the preloaded imaging resources from picture archiving and communication system of our hospital were linked automatedly to the radiological ID of each patient on that list. There would be a little lag to fetch the radiological report and specific images by using this hyperlink and preloaded cache. During the MDTM, the electronic checklist was utilized to visualize essential clinical data, particularly radiographic and endoscopic images, for making treatment plans or filtering cases on demand.

### General multidisciplinary approach

In general, a weekly MDTM was held on Monday and started at 9:30 AM. During the meeting, the core members including GI surgeons, a radiologist, a pathologist, a radiation oncologist, and an anesthesiologist on demand would discuss all cases and make a unique treatment plan for each case. Included cases were intentionally classified as three types according to the complexity of cancer disease: general, moderate, and complicated case, respectively. General cases, which had resectable tumors and negative lymph node metastasis during the clinical assessment, would be simply reviewed, and routine surgical procedures would be commonly followed after meetings. Moderate cases, which had suspicious lymph node metastasis or adjacent organ involvement due to tumor progression, would be carefully reviewed; after that, neoadjuvant therapies (chemotherapy, radiotherapy, combined therapies, etc.) or surgery would be considered according to personal details. Complicated cases, which had serious comorbidities, several times of operation, resectable distant metastasis, emergency complications, or specific indications for any surgical intervention, would be discussed more seriously, with the first priority as the sequence of cancer type. Afterward, consecutive treatment plans might be scheduled for those patients.

During the discussion, a clinical tumor stage was assigned to each patient and finally revised by joined specialists based on the 7th edition of TNM staging system from the American Joint Committee on Cancer (AJCC) [15]. This clinical TNM stage (cTNM) was recorded by our secretary (Chen D.), along with a scheduled treatment plan documented for future clinical audit. Routinely, individual treatment plans should be approved by all experts, with major contents noted in medical records. After the meeting, all decisions were stored in our database and subsequently compared with practical treatment and pathological tumor stage (pTNM). Notably, some cases required repeated discussion along the care pathway, and they were considered as new cases once readmitted to our center.

### Study outcomes

During the MDTM, the time spent on the discussion and the amount of reviewed cases were recorded each time. The agreement of tumor stage and therapeutic measures between the MDTM and pragmatic clinical practice was documented by our secretary every 2 weeks. The primary outcome was the improved efficiency of multidisciplinary procedures by using the novel electronic technique in management of GI malignancy. The secondary outcomes included accuracy of clinical staging, length of stay (LOS), and complications associated with any treatment after admission.

### Statistical analyses

Descriptive statistics were reported as means ± standard deviation (SD) if not otherwise indicated. The accuracy of tumor stage was depended upon the percentage of a correct TNM stage from the MDTM in accordance with the final pathologic report. Continuous variables were compared between both groups using Student’s *t* test or 2-sample Mann-Whitney test, with categorical variables compared using *Chi-square* test or Fisher’s exact test where appropriate. All data were managed and analyzed by using the IBM® SPSS® Statistics (Ver. 23.0; Chicago, IL). Two-tailed tests were used, with *P* values < 0.05 regarded as statistically significant.

## Results

### Clinicopathological characteristics

From January 2015 to December 2016, a total of 4057 patients were retrospectively reviewed from our database, with 383 patients were not eligible for this study. In sum, 3674 patients, who participated in the MDTM and followed their treatment plans, were included for the final analysis. The median age of this cohort was 42 (range, 18–92) years, with 75.0% of male adults included. The demographic and clinical characteristics of included patients are summarized in Table 1. Briefly, 2156 (58.7%) patients receiving our novel MDT approach were assigned to the eMDT group, with the rest of 1518 (41.3%) patients allocated to the cMDT group. Comparisons in age (*P =* 0.529), gender (*P =* 0.844), cancer type (*P =* 0.218), comorbidity (*P =* 0.695), treatment plan (*P =* 0.737), and pathological cancer stage (*P =* 0.098) were not significant between two groups. Additionally, the overall number of meetings was similar in both groups. However, the average number of reviewed cases in each month was significantly larger in the eMDT group than that in the cMDT group (172.3 vs. 126.6; *P =* 0.004). Interestingly, the percentage of patients assigned as early stage (stage < III) was relatively decreased in the eMDT group compared with the cMDT group (55.2 vs. 59.2%; *P =* 0.018). Nevertheless, the distribution of pathological stages (early and advanced stages) was identical between both groups (56.8 vs. 54.1%; *P =* 0.098).

**Table 1:** Demographic and clinical characteristics of included patients during MDT discussion

	eMDT group (*n* = 2156)	cMDT group (*n* = 1518)	*P* value
Including period	01/01/16–31/12/16	01/01/15–31/12/15	
Age, years	55.4 ± 16.5	55.7 ± 16.5	0.529
Gender, male to female	1617:539	1138:380	0.844
BMI, kg/m^2^	20.3 ± 6.1	20.8 ± 6.5	
Comorbidity, *n*(%)			0.695
HTN	465(21.6)	310 (20.4)	
DM	289 (13.4)	193 (12.7)	
Others^a^	86 (4.0)	59 (3.9)	
Cancer type, *n*(%)			0.218
Esophageal cancer	159 (7.4)	102 (6.7)	
Gastric cancer	640 (29.7)	419 (27.6)	
Colorectal cancer	1030 (47.8)	756 (49.8)	
GIST	79 (3.7)	73 (4.8)	
Others^b^	332 (15.4)	209 (13.8)	
Meeting times, *n*	45	46	NA
Cases, *n*/month	172.3 ± 31.5	126.6 ± 42.7	0.004
Treatment plan, *n*(%)			0.737
Neoadjuvant therapy + surgery	305 (14.1)	205 (13.5)	
Surgery	1583 (73.4)	1131 (74.5)	
Palliative care	270 (12.5)	182 (12.0)	
cTNM stage, *n*(%)			0.018
I/II	1190 (55.2)	898 (59.2)	
III/IV	966 (44.8)	620 (40.8)	
pTNM stage, *n*(%)			0.098
I/II	1226 (56.8)	821 (54.1)	
III/IV	930 (43.2)	697 (45.9)	

*P* value < 0.05 indicates significant difference

*eMDT* electronic list-based MDT group, *cMDT* conventional MDT group, *BMI* body mass index, *HTN* hypertension, *DM* diabetes mellitus, *GIST* gastrointestinal stromal tumor, *NA* data not available

^a^Other diseases include chronic obstructive pulmonary disease, atherosclerosis, and chronic liver or renal disease

^b^Other cancers, such as pancreatic carcinoma, hepatic cancer, abdominal adenocarcinoma and peritoneal mesothelioma, were reviewed at the MDT meeting. Data are expressed as number (%) or as means ± SD, where appropriate

### Efficiency analysis of MDTM

**Fig. 3:**
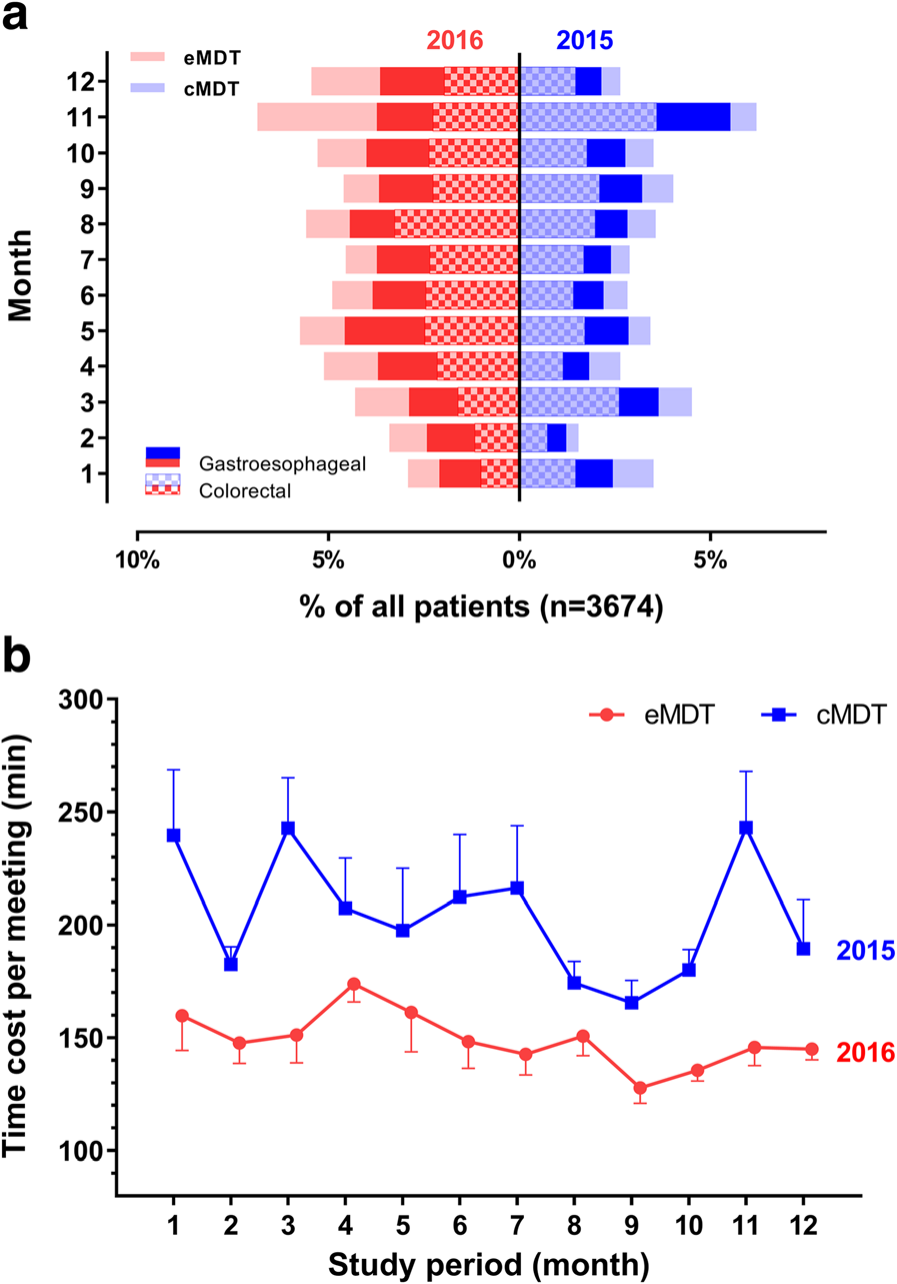
Frequency and time cost comparisons in each month between two groups. **a** The percentage of discussed cases in each sequential month was compared between eMDT and cMDT groups, with subgroups of gastroesophageal and colorectal cancers compared meanwhile. **b** The average time cost in each sequential month was compared between eMDT and cMDT groups

The efficiency of MDT discussion was evaluated with the average time cost in each meeting or each case, with the overall amount of reviewed cases within a certain period of time also considered (Table 2). There were 2289 (62.3%) general cases, 1005 (27.4%) moderate cases, and 380 (10.3%) complicate cases. The case complexity was not significantly different between eMDT and cMDT groups (*P =* 0.070). However, the average time cost in both each meeting and each case were significantly shortened in the eMDT group compared with the cMDT group (*P <* 0.001). Besides, the average amount of monthly reviewed cases was significantly increased in the eMDT group compared with the cMDT group, while the average time cost in each month was markedly reduced (Fig. 3).

**Table 2:** Efficiency analysis of MDT working through two different techniques

	eMDT group (*n* = 2156)	cMDT group (*n* = 1518)	*P* value
Including Period	01/01/16–31/12/16	01/01/15–31/12/15	
Case complexity, *n*(%)			0.070
general	1356 (62.9)	933 (61.5)	
moderate	598 (27.7)	407 (26.8)	
complicate	202 (9.4)	178 (11.7)	
Cases per month, *n*	172.3 ± 31.5	126.6 ± 42.7	0.004
Cases per meeting, *n*	47.9 ± 21.1	33.0 ± 9.5	< 0.001
Time cost per meeting, min	149.4 ± 15.1	205.1 ± 32.7	< 0.001
Time cost per case, min	3.1 ± 3.3	6.2 ± 4.9	< 0.001
Case type			
general	2.6 ± 1.6	4.5 ± 3.7	< 0.001
moderate	3.8 ± 2.2	5.9 ± 3.1	< 0.001
complicate	6.6 ± 3.8	7.2 ± 3.7	0.121
Cancer type			
Esophageal	3.7 ± 2.9	4.2 ± 4.0	0.244
Gastric	3.6 ± 3.1	6.6 ± 4.2	< 0.001
Colorectal	3.0 ± 2.2	6.2 ± 4.7	< 0.001
Others	3.1 ± 2.7	5.8 ± 3.9	< 0.001

*P* value < 0.05 indicates significant difference

Data present as means ± SD if no otherwise indicated

*eMDT* electronic list-based MDT group, *cMDT* conventional MDT group

Further analysis showed that the average time cost in discussing complicated cases was relatively equal with both groups (6.6 vs. 7.2 min; *P =* 0.121), but the percentage of those cases in such cohort was too small to make up the difference from discussing general and moderate cases (Table 2). Additionally, the mean time cost in discussing cases with esophageal cancer was almost identical to both groups (3.7 vs. 4.2 min; *P =* 0.244), but markedly different in discussing cases with other GI cancers (*P <* 0.001; Table 2). Moreover, the average lag time to wait for the loading of radiological images was reduced by 50% with this novel technique, as compared to the conventional method (1.4 vs. 2.7 min; *P <* 0.001).

### Short-term outcomes of included patients

In this cohort, a definitive surgery was performed in 3224 (87.6%) patients, with a neoadjuvant therapy applied in 510 (13.9%) patients. Among those surgical patients, laparoscopic operation was more frequently performed in the eMDT group than that in the cMDT group (65.7 vs. 60.6%; *P =* 0.004). Of note, palliative care was provided for 452 (12.4%) patients due to a confirmed metastatic disease after admission. There was no significant difference in the treatment plan between both groups (*P =* 0.737). However, the overall accuracy of tumor staging by using the electronic list-based MDT approach was relatively improved compared with the traditional MDT fashion (83.0 vs. 74.9%; *P =* 0.070), with statistical significance observed for stage II and III respectively (*P <* 0.001; Table 3). Meanwhile, the average LOS in hospital, as well as postoperative stay, was remarkably reduced in the eMDT group compared with the cMDT group (*P <* 0.001).

**Table 3:** Short-term outcomes of all patients following various multidisciplinary approaches

	eMDT group (*n* = 2156)	cMDT group (*n* = 1518)	*P* value
Including Period	01/01/16–31/12/16	01/01/15–31/12/15	
Accuracy in tumor staging^a^, ratio(%)			0.070
Stage I	326/372 (87.6)	231/266 (86.8)	0.810
Stage II	699/854 (81.8)	386/555 (69.5)	< 0.001
Stage III	472/620 (76.1)	289/450 (64.2)	< 0.001
Stage IV	292/310 (94.2)	231/247 (93.5)	0.859
Overall LOS, days	12.0 ± 7.5	15.1 ± 10.4	< 0.001
Esophageal cancer	15.5 ± 6.8	18.7 ± 9.4	< 0.001
Gastric cancer	16.1 ± 8.4	20.3 ± 10.3	< 0.001
Colorectal cancer	10.4 ± 3.1	13.8 ± 8.3	< 0.001
Other malignancies	7.3 ± 10.2	8.6 ± 11.8	0.161
Overall LOPS, days^b^	8.1 ± 7.2	10.8 ± 8.7	< 0.001
Esophageal cancer	10.2 ± 6.9	12.9 ± 7.2	0.003
Gastric cancer	11.5 ± 5.2	13.8 ± 6.9	< 0.001
Colorectal cancer	6.3 ± 4.1	9.8 ± 5.7	< 0.001
Other malignancies	6.1 ± 9.2	7.8 ± 9.3	0.031
Complications, *n*(%)	383 (17.6)	293 (19.3)	0.243
Bowel obstruction	206 (9.6)	147 (9.7)	0.910
SSI	125 (5.8)	90 (5.9)	0.887
IAH	28 (1.3)	26 (1.7)	0.331
Others^c^	43 (2.0)	31 (2.0)	0.906
Unplanned reoperation, *n*(%)	21 (1.0)	16 (1.0)	0.867

*LOS* length of stay, *LOPS* length of postoperative stay, *SSI* surgical site infection, *IAH* intra-abdominal hemorrhage

^a^Percentage of correct stage in clinical staging as the pathological stage

^b^Patients receiving definitive operation were included for group comparison, with 1888 and 1336 cases in eMDT and cMDT groups respectively

^c^Other complications include fever, pulmonary infection, deep vein thrombosis, urinary tract infection and so on. Data are expressed as number (%) or means ± SD, where appropriate

Complications associated with cancer therapies were recorded in 676 (18.4%) patients, with 35 (0.9%) death cases reported in the current cohort. The incidence of complications in hospital or within 30 days after admission was slightly decreased in the eMDT group compared with the cMDT group (17.6 vs. 19.3%; *P =* 0.243). Unplanned reoperation was performed in 37 (1.0%) patients due to severe postoperative complications, with no significant difference in the reoperation rate between both groups (Table 3; *P =* 0.867).

## Discussion

In this study, the efficiency of our novel MDT method was evaluated in patients with confirmed GI malignancies, with a traditional MDT meeting utilized as control. The findings indicated that the electronic list-based discussion could apparently reduce the time cost in reviewing general and moderate cases. The results also suggested that this novel technique was associated with improved accuracy in clinical tumor staging and reduced hospital stay. Taken all together, it seems that the electronic list-based approach could improve treatment efficiency for such population.

At present, patients with GI malignancies comprise a large portion of all cancer patients, particularly in East Asian countries [16, 17]. In clinical practice, those patients are commonly merged into one group for clinical assessment and discussion, since diagnostic protocols and treatment strategies are similar to most of GI cancers. As a result, we put selected cases with various GI cancers together for subsequent analyses. A recent systematic review also used pooled patients with various GI malignancies to confirm a responsible role of MDT meeting for changes in management of those diseases [13].

Generally, an MDT discussion is essential to achieve the best care of cancer patient [4, 18]. Over the last two decades, a quick development of multimodal therapies for GI malignancy further emphasizes the need of a multidisciplinary approach [19]. Accordingly, more medical specialists convene to review pathology and radiographic imaging, assess clinical tumor stage, and discuss diagnoses and treatment plans for patients. As a return, the accuracy of diagnosis has been greatly improved since more diagnostic protocols used to exclude potential biases [20, 21]. However, improved outcomes, such as survival, have little evidence to confirm yet [4, 18]. In this study, the average overall and postoperative LOS were both distinctively reduced after employing the modified MDT approach, with 3 days shortened averagely compared to the traditional managing method. To our knowledge, the improved understanding of minimally invasive surgery and enhanced recovery after surgery, improvement of surgeons’ skills, and update of surgical equipment may contribute to such finding, with an unclear role of eMDT discussion due to limited data in this area.

Actually, a conventional MDT meeting has to utilize considerable time, efforts, and financial resources [5, 22]. It becomes impracticable due to more newly diagnosed GI cancers [23]. In our center, we have to spend at least 3 to 4 h on the discussion processes for weekly admitted cases by using this traditional approach. Worse, the scheduled treatment plan cannot be fully implemented due to several individual reasons, with additional meetings required for specific cases. Therefore, there is increasing interest in improving the efficiency of MDTM to avoid inappropriate use of time or health resources, while ensuring guaranteed quality for each case. An online-survey study suggests that prioritizing cases or managing some low-risk cases first according to previously agreed protocols might be helpful to improve efficiency and efficacy of MDT discussion.

Our novel technique by using automatic actions of electronic checklist could have three advantages over the traditional approach. First, it totally removes repeated works of fetching history and test results of each case before the meeting. Hence, surgeons being in charge of admitted patients could spend more time in communicating with each patient and gather more details to evaluate case complexity. Second, it allows free sorts of all cases by several conditions, such as cancer type, age, case complexity, and recruited clinical trial. As a result, experts could focus on their specialized fields when dealing with a certain group of patients. At last, it reduces the lag time in loading radiographic images with a cache technique. The saved fragmented time from this novel method could become more apparent when increasing the amount of enrolled cases.

The major limitations of this study need to be mentioned. First, this study is a retrospective analysis, so the study sample has to be selected from available cases in our database. As a result, selection bias could not be avoidable, and the statistical power of current results might be depressed by several confounding factors, such as primary disease, quality of images, and physician characteristics. Second, patients with various cancers in GI tract are put together for analysis. Therefore, the efficiency of this novel technique in treatment of specific cancer remains unknown. A detailed subgroup analysis with sufficient sample size should be required to better understand its role in improving the processes of MDT working. Third, the current study is the absence of long-term results, such as overall survival, disease-free survival, or recurrence-free survival. Hence, whether or not an improved efficiency in management of GI cancers using this novel technique achieves prolonged survival benefit is still suspended. A more strictly designed study with randomized controls and long-term observation is needed to support the present findings.

## Conclusions

This study indicated that the novel MDT discussion based on an electronic checklist could save lots of time in reviewing and discussing cases with various GI cancers. Indirectly, this intelligent technique could increase the capacity of hospital admission and in-patient care at a given time. However, its role in affecting long-term outcomes of such patients remains to be defined by future works.

## Abbreviations

AJCC: American Joint Committee on Cancer
GI: Gastrointestinal
GIST: Gastrointestinal stromal tumor
LOS: Length of stay
MDT: Multidisciplinary team
MDTM: Multidisciplinary team meeting
SD: Standard deviation
